# Phase Diagram and Quantum Entanglement Properties of a Pentamer *S* = 1/2 Heisenberg Spin Cluster

**DOI:** 10.3390/molecules28176418

**Published:** 2023-09-03

**Authors:** Karol Szałowski

**Affiliations:** Department of Solid State Physics, Faculty of Physics and Applied Informatics, University of Łódź, ul. Pomorska 149/153, PL90-236 Łódź, Poland; karol.szalowski@uni.lodz.pl

**Keywords:** quantum entanglement, Wootters concurrence, Heisenberg model, spin cluster, exact diagonalization, canonical ensemble

## Abstract

Cluster molecular magnets prove their potential for applications in quantum technologies, encouraging studies of quantum entanglement in spin systems. In the paper we discuss quantum entanglement properties of pentamer cluster composed of spins S=1/2 forming a tetrahedron with additional spin in its center, with geometry reproducing the smallest nonplanar graph. We model the system with isotropic Heisenberg Hamiltonian including external magnetic field and use exact diagonalization approach to explore the ground-state phase diagram and thermodynamic properties within canonical ensemble formalism. We focus the interest on two-spin entanglement quantified by Wootters concurrence. For ground state, we find two states with total cluster spin equal to 3/2 exhibiting entanglement, occurring preferably for antiferromagnetic interactions. For finite temperatures, we predict the presence of magnetic-field-induced entanglement as well as temperature-induced entanglement.

## 1. Introduction

Molecular magnetism constitutes a flourishing field of study within the theoretical and experimental condensed matter physics and chemistry [1,2], combining the rich fundamental physics of quantum low-dimensional magnets [3,4] with prospective applications in crucial areas such as magnetocaloric cooling [5,6]. However, another factor stimulating research in this scope is the high usefulness of molecular magnets for information storage and processing [7,8,9], both on classical [10] and quantum level [11,12,13,14,15]. The latter revokes the phenomenon of quantum entanglement [16,17,18,19,20].

The interest in quantum entanglement brings the localized spin systems of cluster geometry to the attention of the theoreticians [21,22,23,24]. In such context, a variety of geometries can be mentioned, like dimers [25,26,27,28,29,30,31,32,33,34,35,36], trimers [37,38,39,40,41,42,43] or four-site structures [44,45,46,47,48,49,50,51,52]. Attention is paid to design of cluster structures offering advantageous properties due to interplay between geometry and spin-spin interactions [53,54]. Moreover, the interest can be generalized to entanglement in nanochains of various lengths housing itinerant electrons, with emphasis on both spin and charge degree of freedom [55,56,57,58]. In addition, several experimental works on localized spin systems aimed at capturing the entanglement in zero-dimensional structures have been reported [59,60,61,62,63], supplemented with works focused on systems with higher dimensionality [64,65,66].

Among various shapes of clusters, a pentamer geometry can be highlighted. Such clusters attracted so far some attention of theorists, but only in the context unrelated to quantum entanglement, to mention the neutron scattering simulation [67,68], magnetocaloric properties [69] or general thermodynamics [70]. The experimental studies of usual magnetic properties of these structures were conducted as well [71,72,73]. Within the group, a pentamer formed by inserting a central ion into a tetrahedron can be singled out as geometrically interesting due to high symmetry of its structure and the fact that it constitutes a smallest non-planar graph. Its triangle-based geometry presages considerable magnetic frustration linked to the possible presence of degenerate ground states in the case of competing interactions. Such structures can be found in metal-organic compounds in which Co ions contribute localized spins [74], with a single Co ion possessing octahedral coordination environment and four other ions with tetrahedral environment. Let us also notice that pentanuclear structural units of similar type, tetrahedral in shape and containing Co or Ni ions, were found to build diamond-like networks [75]. Similar pentamers, called diamondoids, were also discussed in Fe-based compounds [76]. What is even more important, an alike structure was found in Cu ion-based compound, where Cu ions contributed localized spins S=1/2 [77]. This particular sort of pentameric structures was subject of some model theoretical studies limited to determining the eigenenergies [78]. Therefore, the quantum entanglement properties of these structures remain unexplored.

Motivated by the synthesability of pentamer spin clusters described above in the field of molecular magnetism and by their non-trivial, highly symmetric geometry, we present a theoretical study focused on characterization of quantum entanglement in such structure. In order to capture the maximized quantum effects, we select cluster composed of spins S=1/2. We explore the full phase diagram of the system in question and identify its possible ground states, considering then ground-state and finite-temperature properties of two-spin entanglement. In the following part of the paper we describe the theoretical model and its thermodynamic solution. Next, we discuss the analytic and numerical results concerning the entanglement in the context of system phase diagram.

## 2. Theoretical Model and Its Thermodynamics

**Figure 1:**
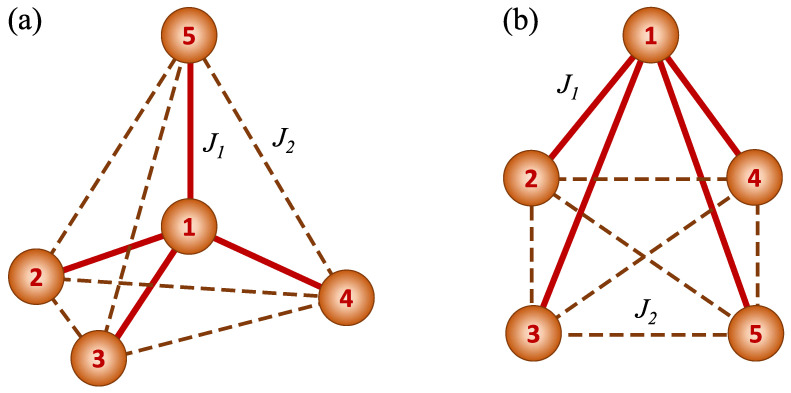
Schematic views of the considered spin cluster with exchange integrals of two kinds marked with different lines: $J_{1}$ with solid lines and $J_{2}$ with dashed lines: (**a**) a tetrahedral structure for spin tetramer with additional spin in the center; (**b**) a planar structure for the tetramer with additional spin interacting with all its members. The numbers from 1 to 5 label the spins.

The system of interest is a spin pentamer cluster, composed of spins S=1/2 forming a regular tetrahedron with an additional spin placed inside it. The schematic view of the considered pentamer cluster is shown in Figure 1a, with two exchange integrals, $J_{1}$ and $J_{2}$, denoted with solid and dashed lines, respectively. The interactions between the spins forming an external tetrahedron (labelled with 2, 3, 4 and 5) are quantified by $J_{2}$, whereas the coupling of all the tetrahedron ions with a central spin (labelled with 1) is equal to $J_{1}$. An alternative structure with equivalent interactions is shown in Figure 1b, composed of a planar tetramer with additional spin. Let us mention, however, that in the structure shown in Figure 1a the distances between the tetramer spins coupled with $J_{2}$ are equal, which is not the case for the structure shown in Figure 1b.

An interesting feature of the studied cluster is that it forms a smallest nonplanar Kuratowski graph, being a complete graph of 5 vertices $K_{5}$ [79], each vertex containing a spin and each spin interacting with each one. As a consequence, there are only two kinds of spin pairs - those connected by $J_{1}$ or $J_{2}$ exchange integrals - and all of them are of nearest-neighbour type. Such non-planarity of the graph representing the spin cluster was also mentioned in other studies on metal-organic pentamers [80,81].

We model the system with isotropic Heisenberg Hamiltonian:

(1) $$\begin{array}{cc} \hat{H}= & -J_{1}{\hat{S}}_{1}\cdot \left({\hat{S}}_{2}+{\hat{S}}_{3}+{\hat{S}}_{4}+{\hat{S}}_{5}\right)-J_{2}\left({\hat{S}}_{2}\cdot {\hat{S}}_{3}+{\hat{S}}_{2}\cdot {\hat{S}}_{4}+{\hat{S}}_{2}\cdot {\hat{S}}_{5}+{\hat{S}}_{3}\cdot {\hat{S}}_{4}+{\hat{S}}_{3}\cdot {\hat{S}}_{5}+{\hat{S}}_{4}\cdot {\hat{S}}_{5}\right)\\ \; & -H\left({\hat{S}}_{1}^{z}+{\hat{S}}_{2}^{z}+{\hat{S}}_{3}^{z}+{\hat{S}}_{4}^{z}+{\hat{S}}_{5}^{z}\right). \end{array}$$

In the above Hamiltonian, the symbol ${\hat{S}}_{i}\equiv \left({\hat{S}}_{i}^{x},{\hat{S}}_{i}^{y},{\hat{S}}_{i}^{z}\right)$ is a vector of quantum operators of spin S=1/2, localized at site labelled by *i*, moreover, ${\hat{S}}_{i}\cdot {\hat{S}}_{j}={\hat{S}}_{i}^{x}{\hat{S}}_{j}^{x}+{\hat{S}}_{i}^{y}{\hat{S}}_{j}^{y}+{\hat{S}}_{i}^{z}{\hat{S}}_{j}^{z}$. Operators for individual spin projections α=x,y,z are given by ${\hat{S}}_{i}^{\alpha }={\hat{\sigma }}_{i}^{\alpha }/2$, where ${\hat{\sigma }}^{\alpha }$ denotes appropriate Pauli matrix. The external magnetic field is introduced by a Zeeman term parametrized with energy *H*.

It should be noticed that the Hamiltonian (1) commutes with an operator of the square of total spin ${\hat{S}}_{T}^{2}={\left({\hat{S}}_{1}+{\hat{S}}_{2}+{\hat{S}}_{3}+{\hat{S}}_{4}+{\hat{S}}_{5}\right)}^{2}$ as well as the projection of the total spin on *z* axis ${\hat{S}}_{T,z}={\hat{S}}_{1}^{z}+{\hat{S}}_{2}^{z}+{\hat{S}}_{3}^{z}+{\hat{S}}_{4}^{z}+{\hat{S}}_{5}^{z}$. Moreover, it commutes also with an operator of the square of the total spin of the tetrahedron (excluding the central spin labelled with 1 in Figure 1) which we denote with ${\hat{s}}^{2}={\left({\hat{S}}_{2}+{\hat{S}}_{3}+{\hat{S}}_{4}+{\hat{S}}_{5}\right)}^{2}$. Therefore, the Hamiltonian eigenstates can be labelled with the appropriate quantum numbers $S_{T}=1/2,\;3/2,\;5/2$, $S_{T,z}=-S_{T},...,S_{T}$ and s=0,1,2, respectively. As a consequence, the eigenenergies of the Hamiltonian (1) can be written in the following form (utilizing the vector coupling method [82]):

(2) $$E_{S_{T},S_{T,z},s}=-\frac{1}{2}J_{1}S_{T}\left(S_{T}+1\right)-\frac{1}{2}\left(J_{2}-J_{1}\right)s\left(s+1\right)+\frac{3}{8}J_{1}+\frac{3}{2}J_{2}-HS_{T,z}.$$

However, it must be stated that some of the states labelled with three quantum numbers $S_{T},\;S_{T,z},\;s$ are further degenerate.

The symbolic or numerically exact diagonalization of the Hamiltonian matrix provides a set of eigenvectors $|\psi_{n}\rangle$ and eigenenergies $E_{n}$ (where $n=1,\cdots ,N_{s}$ and $N_{s}=2^{5}=32$). The complete set of Hamiltonian eigenvalues and eigenstates is given in the Appendix A in Table A1 and Table A2. We label the states as $|\psi_{S_{T},S_{T,z},s}\rangle$ and use additional superscript if the state is degenerate, which is quite often the case for the studied system.

In the description of system states in our work, the single-spin states with spin up (down) are marked with $|↑\rangle$ ($|↓\rangle$), respectively. For multispin states, the order of the arrows corresponds to increasing order of site labels from 1 to 5. This convention is used in Table A1. In order to facilitate interpretation of multispin states, we decompose them also into linear combinations of products of state of a single spin (labelled with 1) and a states of two spin dimers (one of them including spins 2 and 3 and another one composed of spins 4 and 5—see Figure 1). These dimer states are, if convenient, expressed by so called Bell states [83] possessing the following form:

(3) $$\begin{array}{cc} |\phi^{\pm }\rangle & =\frac{|↑↑\rangle \pm |↓↓\rangle }{\sqrt{2}}\\ |\psi^{\pm }\rangle & =\frac{|↑↓\rangle \pm |↓↑\rangle }{\sqrt{2}}. \end{array}$$

Out of Bell states, the state $|\psi^{-}\rangle$ is a singlet state with dimer spin equal to 0, whereas the remaining three states constitute members of a triplet and correspond to dimer spin of 1. Let us notice that the tetrahedron composed of spins 2, 3, 4 and 5 can be decomposed into two dimers in numerous manners, so that our choice is non-unique and arbitrary. However, all the dimer selections are fully equivalent due to high symmetry of interactions within the tetrahedron. The described convention is used to present the eigenstates in Table A2.

The output of Hamitonian diagonalization can be directly used for analysis of the ground state of the system (at temperature T=0) in the full Hamiltonian parameter space spanned by $J_{1}$, $J_{2}$ and *H*. The thermodynamic description of the system is constructed following the rules of canonical ensemble of statistical physics. Let us put emphasis on the fact that the external magnetic field is directly included in the Hamiltonian (1), so that we deal with a field ensemble [84,85] and the thermodynamic average of the Hamiltonian yields enthalpy, not internal energy.

For T>0, the statistical sum can be calculated as:

(4) $$Z=\mathrm{Tr}\;e^{-\hat{H}/\left(k_{B}T\right)}=\sum_{n=1}^{N_{s}}e^{-E_{n}/\left(k_{B}T\right)},$$

where $k_{B}$ denotes Boltzmann constant. The quantum thermal state of the whole cluster is then given by the density matrix:

(5) $$\hat{\rho }=e^{-\hat{H}/\left(k_{B}T\right)}/Z.$$

From the viewpoint of the purpose of the study, the quantum states of spin pairs are of particular interest. The density matrix describing the state of a pair of spins labelled by ij can be obtained by taking a partial trace from $\hat{\rho }$ over remaining spins, i.e.,

(6) $${\hat{\rho }}_{ij}={\mathrm{Tr}}_{klm}\;\hat{\rho },$$

with k,l,m≠i,j and $i,j,k,l,m\in \left\{1,2,3,4,5\right\}$.

In order to quantify quantum entanglement for pairs of spins ij, Wootters concurrence *C* is recalled [86,87]. For separable states C=0, whereas 0<C≤1 corresponds to entangled state. This quantity is calculated for a general mixed state on the basis of the following formula:

(7) $$C=\max\;\left(0,\sqrt{\lambda_{1}}-\sqrt{\lambda_{2}}-\sqrt{\lambda_{3}}-\sqrt{\lambda_{4}}\right),$$

where $\lambda_{1}\geq \lambda_{2}\geq \lambda_{3}\geq \lambda_{4}$ are the eigenvalues of the matrix ${\hat{\rho }}_{ij}{\hat{\rho }}_{ij}^{\prime }$. The auxiliary matrix ${\hat{\rho }}_{ij}^{\prime }$ corresponds to spin flip of the state and is given by:

(8) $${\hat{\rho }}_{ij}^{\prime }=\left({\hat{\sigma }}^{y}⊗{\hat{\sigma }}^{y}\right){\hat{\rho }}_{ij}^{*}\left({\hat{\sigma }}^{y}⊗{\hat{\sigma }}^{y}\right),$$

where ${\hat{\sigma }}^{y}$ is appropriate Pauli matrix.

For the studied system, due to its high symmetry, only two inequivalent values of concurrence can be found, corresponding either to type 1 spin pairs coupled with an exchange integral $J_{1}$ and containing the central spin (termed $C_{\left(1\right)}$) or to type 2 spin pairs coupled with an exchange integral $J_{2}$ containing only the spins from the tetrahedron (termed $C_{\left(2\right)}$).

## 3. Results and Discussion

The results of analytic or numerically exact calculations based on the formalism sketched above will be discussed in this section. In particular, the ground-state phase diagram will be a starting point for analysis of ground-state entanglement and further investigation of the influence of finite temperature on the system properties.

### 3.1. Ground-State Phase Diagram

**Figure 2:**
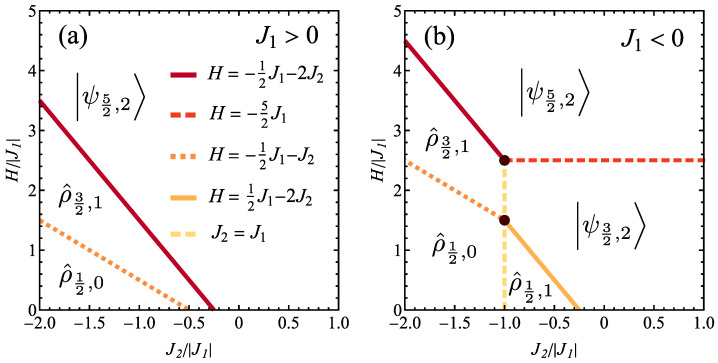
Ground-state magnetic phase diagram of considered spin cluster in the plane $J_{2}/|J_{1}\left|-H/\right|J_{1}|$, for (**a**) ferromagnetic $J_{1}$ and (**b**) antiferromagnetic $J_{1}$ exchange integral. The (linear) phase boundaries are marked with lines and their equations are listed in the inset. The filled circles denote the triple/quadruple points. The pure ground state is denoted by vector $|\psi_{S_{T},s}\rangle$, whereas the mixed (degenerate) ground state is denoted by density matrix ${\hat{\rho }}_{S_{T},s}$.

Let us commence the study of the cluster from the discussion of the ground-state magnetic phase diagram, for T→0. Such diagrams are shown in Figure 2, separately for the case of ferromagnetic interaction $J_{1}>0$ (Figure 2a) and antiferromagnetic interaction $J_{1}<0$ (Figure 2b). In all further discussion, the dimensionless quantities will be used, with $|J_{1}|$ as an unit of energy. Therefore, the cases of $J_{1}>0$ and $J_{1}<0$ will be discussed separately and $J_{1}\neq 0$ is assumed (i.e., the limit of spin tetrahedron without coupling to the central spin is not discussed, as corresponding to two completely uncoupled systems).

It should be emphasized that we deal with a finite spin cluster, therefore, no actual phase transitions are expected and no symmetry breaking phenomenon occurs, contrary to infinite systems in thermodynamic limit. Instead, the diagrams termed as phase diagrams illustrate various ground states of the system and these ground states are not phases in rigorous thermodynamic meaning. However, the term ’phase diagram’ is commonly accepted in this generalized meaning.

Let us notice that due to possible degeneracy of the Hamiltonian eigenstates for the cluster, the ground states for some parameter ranges can be expected to be mixed quantum states (i.e., probabilistic mixtures of degenerate eigenstates with equal probabilities) rather than pure states. Therefore, if we deal with *d* states of the same energy, $|\psi^{\left(q\right)}\rangle ,\;q=1,...,d$, the ground state when T→0 is

(9) $$\hat{\rho }=\frac{1}{d}\sum_{q=1}^{d}|\psi^{\left(q\right)}\rangle \langle \psi^{\left(q\right)}|.$$

If the case of H>0 away from phase boundary is considered, all the states involved must have the same values of all quantum numbers $S_{T}$, $S_{T,z}$ and *s*. The remark about mixed ground state also applies to the case of the phase boundary (i.e., line along which two ground states have the same energy) or a triple/quadruple point (where three/four ground states indicate the same energy) when the degeneracy appears necessarily, even if the neighbouring ground states are pure ones. In both such cases the state of the system is a mixed quantum state being a probabilistic mixture of all the involved Hamiltonian eigenstates sharing the same energy along specific line or at point (this time with different values of the quantum numbers). In the absence of the external magnetic field, for H=0, the additional degeneracy with respect to the value of $S_{T,z}$ emerges [see Equation (2)] and all the states with the same values of $S_{T}$ and *s* share the same energy.

If the ground state for a given range of parameters is non-degenerate, it is marked in Figure 2 for brevity with a ket $|\psi_{S_{T},s}\rangle$ (labelled with the quantum numbers $S_{T}$ for total spin of the cluster and *s* for spin of tetrahedron). In the presence of the external magnetic field H>0 (even arbitrarily weak), the ground state always contains the Hamiltonian eigenstates for which $S_{T,z}=S_{T}$. Therefore, it is sufficient to label the ground states in Figure 2 with just $S_{T}$ and *s*. On the contrary, in the case of a mixed state, it is described by a density matrix ${\hat{\rho }}_{S_{T},s}$ (labelled with the same numbers, as the states degenerate in energy share the same $S_{T}$ and *s*).

In both panels of Figure 2, the boundaries at which the energies corresponding to two different ground states are equal are marked with lines. The equations describing these lines (phase boundaries) are given in the inset. Moreover, the triple or quadruple points, at which the energies of three or four different ground states are equal are marked with bold circles. Let us remark that we discuss a finite spin cluster, so that the existence of quadruple point does not violate Gibbs phase rule, as we do not deal with phase transitions. Such points can be found in ground-state phase diagrams of spin clusters, see for example Refs. [70,88].

#### 3.1.1. Ferromagnetic $J_{1}>0$

The ground-state phase diagram for ferromagnetic $J_{1}>0$ is shown in Figure 2a. In the whole range of magnetic fields for $J_{2}/\left|J_{1}\right|>-1/4$ and just for $H>-\frac{1}{2}J_{1}-2J_{2}$ otherwise, the system takes the saturated ferromagnetic pure state with $S_{T}=S_{T,z}=5/2$ and s=2, i.e., $|\psi_{5/2,+5/2,2}\rangle$ (see Table A1 and Table A2), having the energy of

(10) $$E=-J_{1}-\frac{3}{2}J_{2}-\frac{5}{2}H.$$

For $\frac{1}{2}J_{1}-J_{2}<H<-\frac{1}{2}J_{1}-2J_{2}$ the ground state is a mixed state ${\hat{\rho }}_{3/2,1}$, being a statistical mixture given by Equation (9) involving d=3 states $|\psi_{3/2,+3/2,1}\rangle$ with $S_{T}=S_{T,z}=3/2$ and s=1, listed in Table A1 and Table A2, all sharing the same energy,

(11) $$E=-\frac{1}{2}J_{1}+\frac{1}{2}J_{2}-\frac{3}{2}H.$$

In this state, the central spin inside the tetrahedron (labelled with 1) takes the pure state $|↑\rangle$.

For $H<\frac{1}{2}J_{1}-J_{2}$ (possible only for $J_{2}/\left|J_{1}\right|<-1/2$), the ground state is a mixed state ${\hat{\rho }}_{1/2,0}$ of the form given by Equation (9), composed of d=2 states $|\psi_{1/2,+1/2,0}\rangle$ with $S_{T}=S_{T,z}=1/2$ and s=0, as listed in Table A1 and Table A2, having the energy equal to:

(12) $$E=\frac{3}{2}J_{2}-\frac{1}{2}H.$$

Also in this state, the central spin inside the tetrahedron (labelled with 1) takes the pure state $|↑\rangle$.

Let us separately mention the form of the ground states exactly at H=0, as under this condition the system symmetry is higher and more degeneracies appear because the state energy is $S_{T,z}$-independent.

If $J_{2}/\left|J_{1}\right|>-1/4$, the quantum states $|\psi_{5/2,S_{T,z},2}\rangle$ with $S_{T}=5/2$ and all possible $S_{T,z}=\pm 5/2,\pm 3/2,\pm 1/2$ share the same energy of $E=-J_{1}-\frac{3}{2}J_{2}$ and correspond to s=2. The ground state is a mixed state expressed by Equation (9) with d=6 and the appropriate states are listed in Table A1 and Table A2.

If the coupling fulfils the condition $-1/2<J_{2}/\left|J_{1}\right|<-1/4$, the ground state is composed of d=12 eigenstates $|\psi_{3/2,S_{T,z},1}\rangle$ sharing the energy equal to $E=-\frac{1}{2}J_{1}+\frac{1}{2}J_{2}$, having $S_{T}=3/2$ and $S_{T,z}=\pm 1/2,\pm 3/2$ as well as s=1, possessing the form given by Equation (9) with the relevant states from Table A1 and Table A2.

Some of the above states, corresponding to $S_{T,z}=+3/2$, contribute to the ground state ${\hat{\rho }}_{3/2,1}$ for H>0.

Finally, if $J_{2}/\left|J_{1}\right|<-1/2$, the mixed ground state has energy equal to $E=\frac{3}{2}J_{2}$ and it takes the form predicted by Equation (9) with d=4 states $|\psi_{1/2,S_{T,z},0}\rangle$ having $S_{T}=1/2$, $S_{T,z}=\pm 1/2$ and s=0, given in Table A1 and Table A2 (note that the states for $S_{T,z}=+1/2$ contribute to the state ${\hat{\rho }}_{1/2,0}$ for H>0.).

#### 3.1.2. Antiferromagnetic $J_{1}<0$

The ground-state phase diagram for antiferromagnetic $J_{1}<0$ is depicted in Figure 2b. If $J_{2}/\left|J_{1}\right|<-1$, the same states as those found for $J_{1}>0$ are present in the diagram. If $J_{2}/\left|J_{1}\right|>-1$, two more quantum states can emerge as ground states. In this range, if the magnetic field exceeds $H/|J_{1}|=5/2$, the saturated ferromagnetic state occurs. However, below this field, but for $H>\frac{1}{2}J_{1}-2J_{2}$, a pure state with $S_{T}=S_{T,z}=3/2$ and s=2, $|\psi_{3/2,+3/2,2}\rangle$, is a ground state of the system (see Table A1 and Table A2); it has energy equal to

(13) $$E=\frac{3}{2}J_{1}-\frac{3}{2}J_{2}-\frac{3}{2}H.$$

In this state, the central spin inside a tetrahedron, labelled with 1, is in mixed state of the form $\frac{1}{5}|↑\rangle \langle ↑|+\frac{4}{5}|↓\rangle \langle ↓|$.

If $H<\frac{1}{2}J_{1}-2J_{2}$ (only for $J_{2}/\left|J_{1}\right|<-1/4$), the ground state is mixed state ${\hat{\rho }}_{1/2,1}$ [see Equation (9)] composed of d=3 states with $S_{T}=S_{T,z}=1/2$ and s=1, listed in Table A1 and Table A2 as $|\psi_{1/2,+1/2,1}\rangle$, sharing the energy of

(14) $$E=\frac{1}{2}J_{1}+\frac{1}{2}J_{2}-\frac{1}{2}H.$$

In this state, the spin labelled with 1 is in mixed state of the form $\frac{1}{3}|↑\rangle \langle ↑|+\frac{2}{3}|↓\rangle \langle ↓|$.

Again, for H=0 the degeneracy of the Hamiltonian eigenvalues increases significantly and the ground states are discussed separately.

If $J_{2}/\left|J_{1}\right|>-1/4$, the ground state is a mixed state [Equation (9)] containing d=4 eigenstates $|\psi_{3/2,S_{T,z},2}\rangle$ of energy $E=\frac{3}{2}J_{1}-\frac{1}{2}J_{2}$, with $S_{T}=3/2$, $S_{T,z}=\pm 3/2,\pm 1/2$ and s=2, taking the form indicated in Table A1 and Table A2.

In the case of $-1<J_{2}/\left|J_{1}\right|<-1/4$, the ground state [Equation (9)] is formed out of d=6 eigenstates $|\psi_{1/2,S_{T,z},1}\rangle$ with energy $E=J_{1}+\frac{1}{2}J_{2}$ and $S_{T}=1/2$, $S_{T,z}=\pm 1/2$ and s=1, which are expressed in Table A1 and Table A2.

Finally, if $J_{2}/\left|J_{1}\right|<-1$, the quantum state is a mixed state discussed already for ferromagnetic $J_{1}>0$, possessing the energy of $E=\frac{3}{2}J_{2}$ (it is notable that the energy is independent on $J_{1}$) and taking the form predicted by Equation (9) with d=4 states $|\psi_{1/2,S_{T,z},0}\rangle$ having $S_{T}=1/2$, $S_{T,z}=\pm 1/2$ and s=0, of the form expressed in Table A1 and Table A2.

After analysis of the ground-state phase diagram it can be concluded that relatively large area is filled with states showing degeneracy, due to high symmetry of the studied cluster.

### 3.2. Ground-State Entanglement

**Figure 3:**
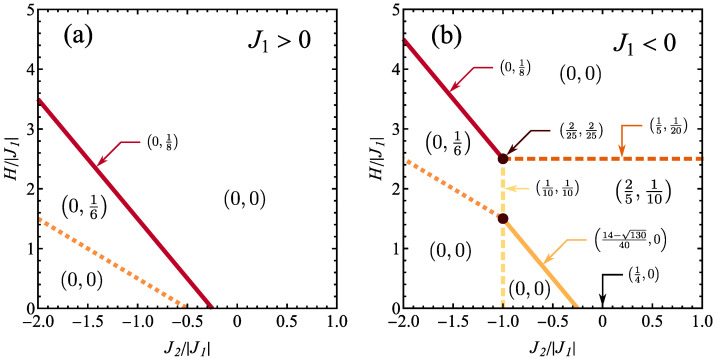
Ground-state phase diagram showing the values of Wootters concurrences $\left(C_{\left(1\right)},C_{\left(2\right)}\right)$ for spin pairs interacting with $J_{1}$ and $J_{2}$ exchange integral, respectively (see Figure 1), for (**a**) ferromagnetic $J_{1}$ and (**b**) antiferromagnetic $J_{1}$ exchange integral. The color lines mark the phase boundaries as in Figure 2. The values of concurrences exactly along the phase boundaries or at the triple point are shown with arrows. If no arrow points to the phase boundary or a point, both concurrences vanish there.

The system ground states can exhibit entanglement for the case of ferromagnetic $J_{1}$ (Figure 3a) and of antiferromagnetic $J_{1}$ (Figure 3b); both cases are analysed in the further part of the paper.

In the context of the ground state being a mixed state given by Equation (9) for the case of degeneracy, it should be mentioned that the creation of probabilistic mixture of entangled states may lead to entangled or to separable state. Therefore, even if the member states $|\psi^{\left(q\right)}\rangle$ in Equation (9) are entangled, the state $\hat{\rho }$ is not necessarily entangled. This is an important observation for the case of cluster systems with degeneracy of ground states. Some results regarding this phenomenon were, for example, discussed in Ref. [89], for the case of probabilistic mixture of two pure states. The results discussed in Ref. [89] show the overall complicacy of the relation of the entanglement of pure states and their probabilistic mixture.

The values of the Wootters concurrence for the ground state, at T=0, are shown in Figure 3 in a form of pairs $\left(C_{\left(1\right)},C_{\left(2\right)}\right)$, indicating the concurrence of inequivalent spin pairs of both types, as discussed previously. Apart from the values valid for given phase, the values of concurrence are also assigned to the boundaries between the phases and to triple point, as shown by the arrows. Let us notice here that at the phase boundary or triple point all the Hamiltonian eigenstates corresponding to two or three phases take the same energy, so that the quantum state is a probabilistic mixture of all that states. Moreover, also the concurrence value at H=0 for some range of interactions is indicated with an arrow, as the ground state degeneracy in the absence of the magnetic field is increased and the concurrence value may be different than that achieved for H>0.

It is visible that three ground states are separable: the ferromagnetic saturated spin-5/2 one (given by $|\psi_{5/2,2}\rangle$ (see Table A1 or Table A2) and two states with spin-1/2, given by ${\hat{\rho }}_{1/2,0}$ and ${\hat{\rho }}_{1/2,1}$. As a consequence, only two ground states with spin-3/2 exhibit quantum entanglement.

For the cluster state described by ${\hat{\rho }}_{3/2,1}$, only the spin pairs belonging to the outer tetrahedron are entangled and the concurrence value is 1/6. The form of pair state is:

(15) $$\hat{\rho }=\frac{1}{2}|↑↑\rangle \langle ↑↑|+\frac{1}{4}\left(|↑↓\rangle \langle ↑↓|+|↓↑\rangle \langle ↓↑|\right)-\frac{1}{12}\left(|↓↑\rangle \langle ↑↓|+|↑↓\rangle \langle ↓↑|\right)$$

or

(16) $$\hat{\rho }=\frac{1}{4}\left(|\phi^{+}\rangle \langle \phi^{+}|+|\phi^{-}\rangle \langle \phi^{-}|+|\phi^{+}\rangle \langle \phi^{-}|+|\phi^{-}\rangle \langle \phi^{+}|\right)+\frac{1}{6}|\psi^{+}\rangle \langle \psi^{+}|+\frac{1}{3}|\psi^{-}\rangle \langle \psi^{-}|.$$

It can be mentioned that the state (15) is an example of probabilistic mixture of all four Bell (maximally entangled) states with additional admixture of non-diagonal states. It is visible that the concurrence value is significantly decreased with respect to the component states (as shown, for example, in Ref. [89] for the specific case of two states in probabilistic mixture).

Moreover, exactly at the boundary of this state with saturated ferromagnetic state, weaker entanglement demonstrated by concurrence equal to 1/8 is present. Due to the topology of the phase diagram for the ferromagnetic $J_{1}>0$, the entanglement is present only for a finite range of magnetic fields for $J_{2}$ strong enough and of antiferromagnetic sign, as this is the only spin-3/2 state found for $J_{1}>0$ (see Figure 3a). The same spin-3/2 state is also present in the phase diagram for $J_{1}<0$ (Figure 3b), but its presence is limited to $J_{2}/\left|J_{1}\right|<-1$. For $J_{2}/\left|J_{1}\right|>-1$ it is replaced with a pure spin-3/2 state $|\psi_{3/2,2}\rangle$, for which both kinds of spin pairs are entangled. The pairs involving the central spin indicate quite noticeable concurrence of 2/5 and their state takes the form of:

(17) $$\hat{\rho }=\frac{3}{20}|↑↑\rangle \langle ↑↑|+\frac{1}{20}|↑↓\rangle \langle ↑↓|+\frac{4}{5}|↓↑\rangle \langle ↓↑|-\frac{1}{5}\left(|↓↑\rangle \langle ↑↓|+|↑↓\rangle \langle ↓↑|\right)$$

or

(18) $$\begin{array}{cc} \hat{\rho } & =\frac{3}{40}\left(|\phi^{+}\rangle \langle \phi^{+}|+|\phi^{-}\rangle \langle \phi^{-}|+|\phi^{+}\rangle \langle \phi^{-}|+|\phi^{-}\rangle \langle \phi^{+}|\right)\\ \; & +\frac{9}{40}|\psi^{+}\rangle \langle \psi^{+}|+\frac{5}{8}|\psi^{-}\rangle \langle \psi^{-}|-\frac{3}{8}\left(|\psi^{+}\rangle \langle \psi^{-}|+|\psi^{-}\rangle \langle \psi^{+}|\right). \end{array}$$

The pairs built of tetrahedron (outer) spins are less entangled, with concurrence value of 1/10 and quantum state:

(19) $$\hat{\rho }=\frac{9}{10}|↑↑\rangle \langle ↑↑|+\frac{1}{20}\left(|↑↓\rangle \langle ↑↓|+|↓↑\rangle \langle ↓↑|\right)+\frac{1}{20}\left(|↓↑\rangle \langle ↑↓|+|↑↓\rangle \langle ↓↑|\right)$$

or

(20) $$\hat{\rho }=\frac{9}{20}\left(|\phi^{+}\rangle \langle \phi^{+}|+|\phi^{-}\rangle \langle \phi^{-}|+|\phi^{+}\rangle \langle \phi^{-}|+|\phi^{-}\rangle \langle \phi^{+}|\right)+\frac{1}{10}|\psi^{+}\rangle \langle \psi^{+}|.$$

Let us note that also the boundary of both spin-3/2 states shows the presence of entanglement, as well as the triple point at which two spin-3/2 states and spin-5/2 state have the same energy. Also the boundary between spin-3/2 and saturated ferromagnetic state corresponds to non-vanishing entanglement of both kinds of spin pairs. On the other hand, at the boundary between the pure spin-3/2 state and spin-1/2 state ${\hat{\rho }}_{1/2,1}$ only the first kind pairs are weakly entangled, with concurrence value of $\left(14-\sqrt{130}\right)/40≃0.065$. For completeness, we also mention that for $J_{2}/\left|J_{1}\right|>-1/4$ exactly at H=0, the spin pairs from the tetrahedron exhibit concurrence value of 1/4 and their state is:

(21) $$\hat{\rho }=\frac{1}{8}\left(|↑↑\rangle \langle ↑↑|+|↓↓\rangle \langle ↓↓|\right)+\frac{3}{8}\left(|↑↓\rangle \langle ↑↓|+|↓↑\rangle \langle ↓↑|\right)-\frac{1}{4}\left(|↓↑\rangle \langle ↑↓|+|↑↓\rangle \langle ↓↑|\right)$$

or

(22) $$\hat{\rho }=\frac{1}{8}\left(|\phi^{+}\rangle \langle \phi^{+}|+|\phi^{-}\rangle \langle \phi^{-}|\right)+\frac{1}{8}|\psi^{+}\rangle \langle \psi^{+}|+\frac{5}{8}|\psi^{-}\rangle \langle \psi^{-}|.$$

### 3.3. Finite Temperature Entanglement

The effect of the finite temperature T>0 on the quantum entanglement generally consists in creation of a thermal mixture of all available system states [Equation (5)]. In the regions of phase diagram where the ground state is entangled, this thermal admixture of separable states may tend to reduce the concurrence (and in the limit of T→∞ the thermal state is always separable). Let us notice that the robustness of entanglement in thermal states focused some general attention [90,91,92] and even for the case of two qubits (spins) the thermal behaviour of entanglement can be rich [93]; noticeably, the separability of the ground state does not imply absence of entanglement at finite temperatures (so that a sort of temperature-induced entanglement can emerge, as observed for example in Ref. [25]). Again, it recalls the problem of entanglement of a probabilistic mixture of pure Hamiltonian states according to Equation (5) in canonical ensemble.

The discussion of the finite-temperature properties will first involve the case of $J_{1}>0$ (when only the spin pairs of second kind can be entangled) and then the case of $J_{1}<0$ (when both kinds of spin pairs can exhibit entanglement).

#### 3.3.1. Ferromagnetic $J_{1}>0$

**Figure 4:**
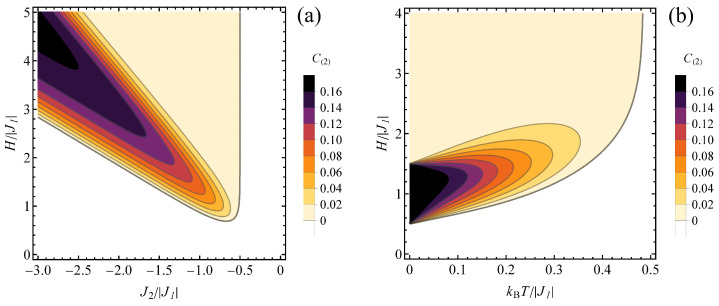
Contour plot of $C_{\left(2\right)}$ concurrence value for $J_{1}>0$: (**a**) at the finite normalized temperature of $k_{B}T/\left|J_{1}\right|=$ 0.2, in the plane $J_{2}/|J_{1}\left|-H/\right|J_{1}|$; (**b**) for $J_{2}/\left|J_{1}\right|=$−1, in the plane $k_{B}T/|J_{1}\left|-H/\right|J_{1}|$. Color bar right to each panel shows the concurrence values for each contour. The white range corresponds to separable states.

The effect of finite temperature on the concurrence values for $J_{1}>0$ can be first tracked in Figure 4a, for spin pairs of second kind, at the normalized temperature of $k_{B}T/\left|J_{1}\right|=0.2$ (compare the ground-state phase diagram in Figure 3a). It is visible that close to the boundaries between the states the entanglement is most effectively reduced (as visible for weakly antiferromagnetic $J_{2}$). This is due to the fact that close to the boundary between the entangled and separable ground state their energy difference is small, so that the coefficients describing the amount of both states in a thermal mixture become comparable. On the other hand, a range of weak entanglement appears for $J_{2}/\left|J_{1}\right|<-1/2$ for the span of stronger magnetic fields without upper critical magnetic field (above which the ground-state would be separable).

**Figure 5:**
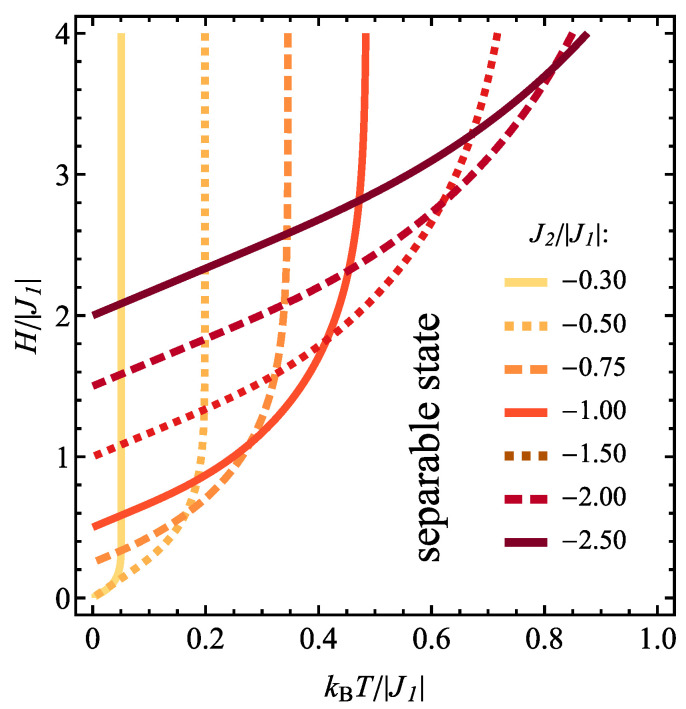
The boundary between the entangled and the separable state of type 2 spin pairs in the plane $k_{B}T/|J_{1}\left|-H/\right|J_{1}|$, for $J_{1}>0$ and for various values of $J_{2}/\left|J_{1}\right|$.

The effect of variable temperature and magnetic field on the concurrence in the case of $J_{1}>0$ can be followed in Figure 4b, in a contour plot prepared for constant $J_{2}/\left|J_{1}\right|=-1$. The influence of the temperature comprises reduction of concurrence and also shifting the critical magnetic field between separable and entangled states to higher values. At finite temperatures, the entanglement is present above the critical magnetic field, exceeding always the value of $H/|J_{1}|=1/2$. This sort of boundary dividing the quantum thermal states to separable and entangled ones can be plotted separately in normalized temperature-normalized magnetic field plane for various values of $J_{2}/|J_{1}$, as it is done in Figure 5. For the values of $J_{2}/\left|J_{1}\right|>-1/2$, the critical magnetic field increases very fast with the temperature, with the value of critical field equal to 0 at T=0. For couplings $J_{2}/\left|J_{1}\right|<-1/2$ the nonzero critical magnetic field for the onset of entanglement occurs; it increases with rising temperature and also with antiferromagnetic coupling $J_{2}$ magnitude.

**Figure 6:**
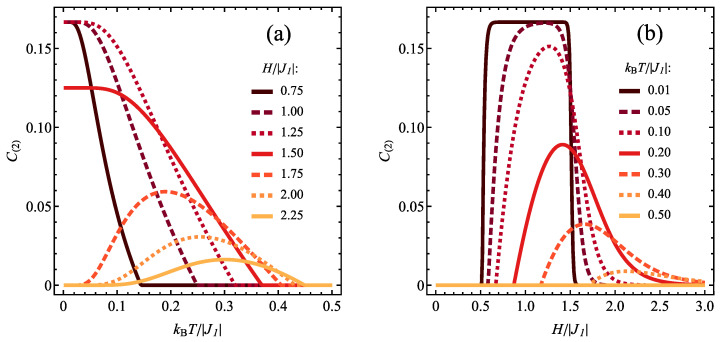
Dependence of the concurrence values $C_{\left(2\right)}$ (**a**) on the normalized temperature, for various values of normalized magnetic field and (**b**) on the normalized magnetic field, for various values of normalized temperature, for $J_{1}>0$ and $J_{2}/\left|J_{1}\right|=$ −1.

The cross-sections of Figure 4b are shown in Figure 6a for various selections of constant magnetic field and variable temperature and Figure 6b for various selections of constant temperature and variable magnetic field. In Figure 6a, for lower magnetic fields, the concurrence decreases monotonically with temperature from the initial, ground-state value of 1/6 characteristic of the state ${\hat{\rho }}_{3/2,1}$. Exactly at $H/|J_{1}|=3/2$ the initial value switches to 1/8 when the state ${\hat{\rho }}_{3/2,1}$ gains statistical admixture of separable saturated ferromagnetic state $|\psi_{5/2,2}\rangle$, but the overall behaviour as a function of temperature is unchanged. On the contrary, for the higher fields, the temperature is a factor causing the emergence of entanglement, which is absent in the ground state ${\hat{\rho }}_{1/2,0}$. For this regime, a maximum of concurrence is formed when *T* increases (due to the thermal admixture of spin-3/2 states), but its height is reduced when the magnetic field rises (which is the factor favouring energetically the saturated spin-5/2 separable state). The effect of the increasing magnetic field can be tracked in Figure 6b. In all studied temperatures the state is separable below certain critical magnetic field, which switches on the entanglement (due to transition to the state with significant participation of the entangled state ${\hat{\rho }}_{3/2,1}$ at finite *T*) and a maximum is formed (with values up to 1/6 for the lowest temperatures, as in ${\hat{\rho }}_{3/2,1}$ state). At higher fields the concurrence tends to vanish gradually as the separable state with spin 5/2 dominates. This is an example of competing influence of temperature and magnetic field on the entanglement. For lower magnetic fields, the magnetic field increases the amount of entangled ${\hat{\rho }}_{3/2,1}$ state in the thermal state, boosting the concurrence, whereas the thermal fluctuations at elevated temperature restore the higher amount of separable ground state ${\hat{\rho }}_{1/2,0}$ lowering the concurrence value. At higher fields the situation is opposite: further increase of the field enriches the thermal state with saturated ferromagnetic separable state reducing the concurrence, whereas the thermal fluctuations at elevated temperature restore the higher amount of entangled state ${\hat{\rho }}_{3/2,1}$ boosting the concurrence value.

#### 3.3.2. Antiferromagnetic $J_{1}<0$

**Figure 7:**
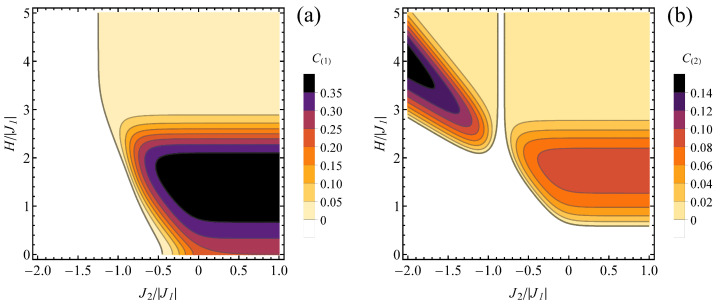
Contour plot of (**a**) $C_{\left(1\right)}$ and (**b**) $C_{\left(2\right)}$ concurrence value at the finite normalized temperature of $k_{B}T/\left|J_{1}\right|=$ 0.2, in the plane $J_{2}/|J_{1}\left|-H/\right|J_{1}|$, for $J_{1}<0$. Color bar right to each panel shows the concurrence values for each contour. The white range corresponds to separable states.

For antiferromagnetic $J_{1}$, both types of spin pairs can exhibit entanglement. A finite temperature ($k_{B}T/\left|J_{1}\right|=0.2$) contour plot of concurrence is shown in the $J_{2}/\left|J_{1}\right|$ − $H/|J_{1}|$ plane in Figure 7, for type 1 (Figure 7a) and type 2 (Figure 7b) spin pairs. Again, it resembles a thermally diffused version of the relevant ground-state phase diagram shown in Figure 3b. For spin pairs connected with $J_{1}$, the most pronounced entanglement is present for the parameters range corresponding to $|\psi_{3/2,2}\rangle$ ground state in Figure 2b, with values reduced close to the boundaries of this range due to thermal admixture of separable states. On the other hand, the thermal state for $J_{2}>0$ and considerably high magnetic field acquires some residual entanglement due to thermal effects (as opposed to separable saturated ferromagnetic ground state). A similar qualitative picture can be tracked in Figure 7b for concurrence in type 2 spin pairs. Both entangled ranges of the phase diagram (Figure 3b) corresponding to ground states with $S_{T}=3/2$ reduce their entanglement and become separated when the temperature is elevated, whereas the saturated ferromagnetic state acquires residual concurrence by admixture of other states.

**Figure 8:**
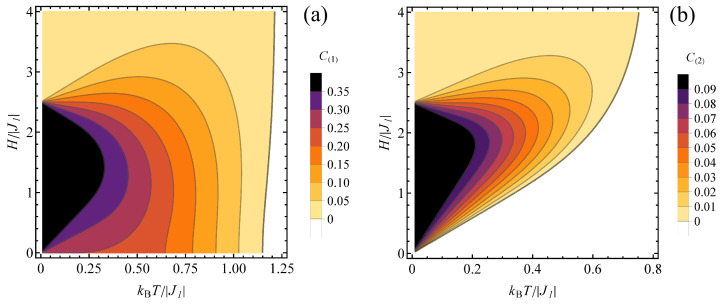
Contour plot of (**a**) $C_{\left(1\right)}$ and (**b**) $C_{\left(2\right)}$ concurrence value for $J_{2}/\left|J_{1}\right|=$ 1 and $J_{1}<0$, in the plane $k_{B}T/|J_{1}\left|-H/\right|J_{1}|$. Color bar right to each panel shows the concurrence values for each contour. The white range corresponds to separable states.

The influence of the temperature and magnetic field on the entanglement for both types of spin pairs can be followed in contour plots in Figure 8, prepared for $J_{2}/\left|J_{1}\right|$ = 1. For type 1 pairs (Figure 8a), the maximum concurrence is achieved for a finite magnetic field which does not shift significantly when the temperature is elevated (and there is no minimum field necessary to trigger the entanglement). Moreover, the boundary dividing the phase diagram into separable and entangled states corresponds to relatively high temperature and is relatively insensitive to the field. For type 2 pairs (Figure 8b), the magnetic field at which the concurrence peaks shifts to higher values when the temperature rises and the entanglement is absent below certain critical field. Contrary to the case of type 1 pairs, the boundary separating the entangled and separable states is linear at lower temperatures and its slope increases for higher values of temperature.

**Figure 9:**
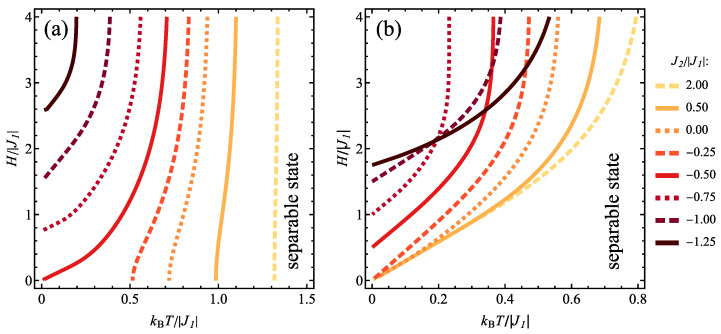
The boundary between the entangled and the separable state of type 1 spin pairs (**a**) and type 2 spin pairs (**b**) in the plane $k_{B}T/|J_{1}\left|-H/\right|J_{1}|$, for $J_{1}<0$ and for various values of $J_{2}/\left|J_{1}\right|$.

The mentioned boundary lines dividing entangled and separable states can be plotted complementarily in temperature-magnetic field plane in Figure 9 for various selected values of $J_{2}/\left|J_{1}\right|$. For type-1 pairs (Figure 9a), the boundary for $J_{2}>0$ is rather weakly dependent on the applied field and the non-zero critical temperature above which the concurrence vanishes decreases when $J_{2}$ coupling tends to 0. Exactly for $J_{2}$ = 0 (i.e., spins belonging to the tetrahedron uncoupled to each other) the boundary commences at T=0 and the critical temperature rises when stronger field is applied. For antiferromagnetic $J_{2}<0$ this tendency remains, but the entanglement is present only above certain critical field which is increased when $J_{2}$ becomes stronger. For type-2 pairs (Figure 9b), for ferromagnetic or weakly antiferromagnetic $J_{2}$, the entanglement is present only at finite magnetic fields and this minimum field increases when the temperature is elevated; in addition also reducing the coupling $J_{2}$ results in increase of the field. For $J_{2}/\left|J_{1}\right|>-1/4$ all the boundaries commence at T=0 and H=0. If the coupling $J_{2}$ is more antiferromagnetic, the critical magnetic field necessary to trigger the entanglement is non-zero even for T=0 and it is increased when the temperature rises (the overall temperature range for which concurrence is positive gets reduced when the antiferromagnetic coupling $J_{2}$ becomes stronger). For even stronger antiferromagnetic $J_{2}$, the temperature dependence of the critical field tends to flatten.

**Figure 10:**
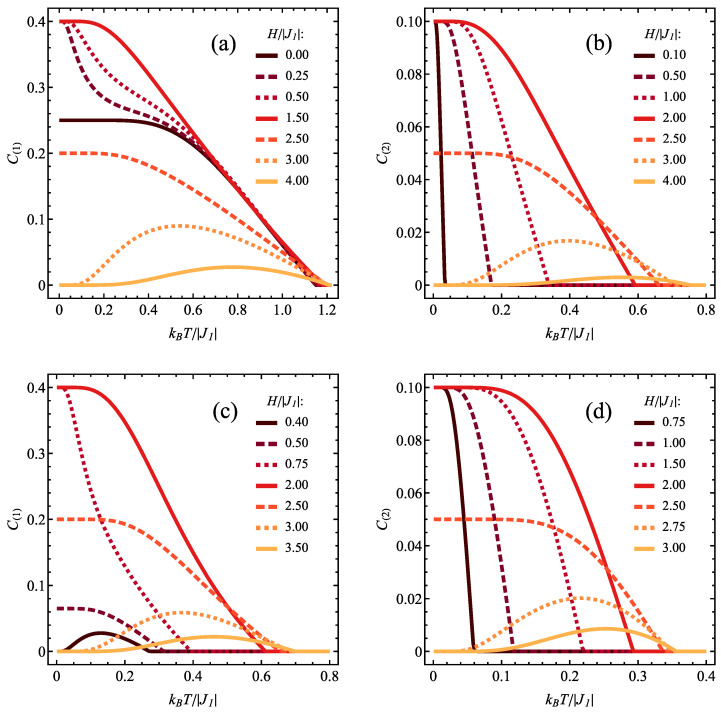
Dependence of the concurrence values $C_{\left(1\right)}$ [for (**a**,**c**)] and $C_{\left(2\right)}$ [for (**b**,**d**)] on the normalized temperature, for various values of normalized magnetic field, for $J_{1}<0$ and for $J_{2}/\left|J_{1}\right|=$ 1 [(**a**,**b**)] or for $J_{2}/\left|J_{1}\right|=$−0.5 [(**c**,**d**)].

The detailed temperature dependence of concurrence for both considered spin pairs for selected magnetic fields can be tracked in Figure 10. The case of ferromagnetic $J_{2}/\left|J_{1}\right|$ = 1 is depicted in Figure 10a for type 1 pairs and Figure 10b for type 2 pairs. For type 1 pairs, in the absence of the field $C_{\left(1\right)}$ takes the ground state value of 1/4 at T=0 (characteristic of probabilistic mixture of states $|\psi_{3/2,S_{T,z},2}\rangle$ with all allowed values of $S_{T,z}$) and monotonically decreases to 0 with increasing slope when the temperature rises (with a long plateau at the lowest temperatures). For H>0, the initial ground-state value of concurrence is equal to 2/5 (as the ground state is $|\psi_{3/2,2}\rangle$), but $C_{\left(1\right)}$ first drops down fast to approximately 1/4 (because for weak field the states $|\psi_{3/2,S_{T,z},2}\rangle$ with all allowed values of $S_{T,z}$ lie close in energy and form probabilistic mixture as thermal fluctuations arise) and then further decreases when *T* is elevated. For stronger fields the temperature dependence of $C_{\left(1\right)}$ becomes more regular. Exactly for $H/|J_{1}|$ = 5/2 the initial value of concurrence is 1/5 (as the mixture of states with spin-3/2 and spin-5/2 is created) with further monotonic decrease. At stronger fields the behaviour changes qualitatively, as the entanglement vanishes at T=0 (as the ground state is saturated ferromagnetic one) and a local maximum builds up when the temperature increases (so that we deal with thermally induced entanglement). For the strongest field the maximum height becomes reduced and its position is shifted to higher temperatures. The behaviour of $C_{\left(2\right)}$ is shown in Figure 10b. For this kind of spin pairs the initial value of concurrence for low temperatures is 1/10 for zero field or low fields (characteristic of ground state $|\psi_{3/2,2}\rangle$), with monotonic behaviour as a function of the temperature. The same sort of behaviour is seen for $H/|J_{1}|$ = 5/2 but with the initial value of 1/20 (as the ground state involves entangled state with spin 3/2 and separable ferromagnetic one with spin 5/2). For stronger fields a temperature-induced local maximum of concurrence is formed, like in the case of type 1 spin pair.

When the coupling $J_{2}$ is antiferromagnetic, the situation is qualitatively different. For type 1 pairs (as shown in Figure 10c) at weak magnetic field the entanglement is only induced by finite temperature, with a local maximum (as the ground state is separable ${\hat{\rho }}_{1/2,1}$ and only the thermal admixture of spin-3/2 state induces the entanglement). When the field increases, the ground-state entanglement emerges (either due to ground state being $|\psi_{3/2,2}\rangle$ or owing to its admixture to other states at phase boundaries) and the temperature dependence takes the form of monotonic decrease (with varying initial values in concert with the diagram in Figure 3b). For even stronger fields the picture switches back to the scenario with temperature-induced local maximum of concurrence (as the ground state becomes saturated separable ferromagnetic state). In the case of type 2 pairs (Figure 10d), the monotonic decrease from the initial value of 1/10 (characteristic of entangled ground state $|\psi_{3/2,2}\rangle$) is noticeable for weaker fields, whereas for stronger ones a local maximum of temperature-induced concurrence is present (and the entanglement is thermally induced by admixing spin-3/2 states to saturated ferromagnetic ground state).

**Figure 11:**
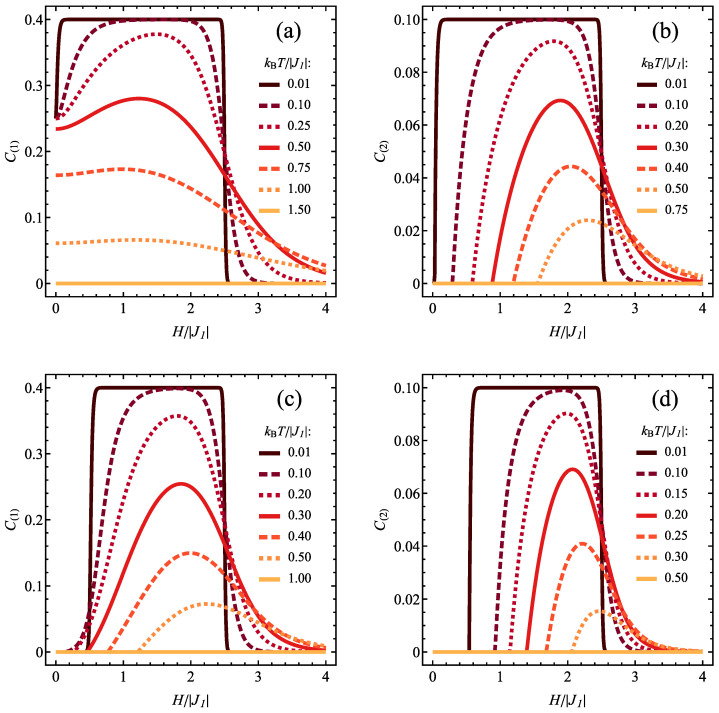
Dependence of the concurrence values $C_{\left(1\right)}$ [for (**a**,**c**)] and $C_{\left(2\right)}$ [for (**b**,**d**)] on the normalized magnetic field, for various values of normalized temperature, for $J_{1}<0$ and for $J_{2}/\left|J_{1}\right|=$ 1 [(**a**,**b**)] or for $J_{2}/\left|J_{1}\right|=$−0.5 [(**c**,**d**)].

Tracking of the detailed magnetic field dependence of concurrence for various temperatures is possible in Figure 11. For type 1 spin pairs and ferromagnetic $J_{2}/\left|J_{1}\right|$ = 1 (Figure 11a), at lower temperatures the concurrence takes in the absence of the field the value of 1/4 (characteristic of mixed state involving $|\psi_{3/2,S_{T,z},2}\rangle$ with all allowed values of $S_{T,z}$, see Figure 3b) and fast jumps to 2/5 (stemming from $|\psi_{3/2,2}\rangle$ ground state), reaching a plateau persisting up to certain critical magnetic field. The increasing temperature first tends to reduce the value at the plateau (as the thermal state acquires contribution from saturated ferromagnetic state) and then also diminishes the initial concurrence value at *H* = 0. At the same time the range of entangled states is extended to higher magnetic fields, as the thermal admixture of entangled spin-3/2 state builds up entanglement in the range where the ground state has spin 5/2. For type 2 pairs (Figure 11b), the entanglement is induced by the finite field *H*. At the lowest temperatures its field dependence reaches the highest plateau with the value of 1/10 (emerging for $|\psi_{3/2,2}\rangle$ ground state), and the increasing temperature smears this maximum, increases a threshold magnetic field for the onset of entanglement and makes the entanglement more robust in the range of higher fields. This behaviour exemplifies again the competing effect of temperature and magnetic field on the entanglement, as discussed in the context of Figure 6b. The magnetic field influence on the concurrence of type 1 spin pairs for antiferromagnetic $J_{2}/\left|J_{1}\right|$ = −0.5 (Figure 11c) is somehow similar as in the previous case (the entanglement is field-induced); however, the maximum corresponds to the values reaching 2/5. The situation illustrated in Figure 11d for type 2 pairs resembles even more the scenario for ferromagnetic $J_{2}$.

## 4. Conclusions

In the paper we have reported computational study of a pentamer spin cluster (with single spin located in the center of spin tetrahedron) composed of spins S=1/2 and described with isotropic Heisenberg model with external magnetic field and two exchange integrals: one for spin pairs belonging to tetrahedron and another one quantifying the coupling between tetrahedron spins and a central spin. The cluster has non-trivial geometry characterized by high symmetry (being the smallest non-planar Kuratowski graph K${}_{5}$). The selected geometry offers the possibility of influencing the ratio of exchange integrals by chemical composition and of creating networks of weakly interacting clusters [75]. The interest of our study was focused on the quantum two-spin entanglement properties. Both ground-state properties and the effect of finite temperature on the system behaviour were discussed, preceded by the analysis of the ground-state phase diagram as a function of exchange integrals and external magnetic field. A specific feature of the selected cluster is relatively frequent occurrence of ground state degeneracy due to high system symmetry. We found the presence of two-spin entanglement for two ground states (a pure one and a mixed one), both with total cluster spin number equal to $S_{T}=3/2$. The studied cluster exhibits the entanglement preferably for all-antiferromagnetic couplings, which is an usual pattern in the case of magnetic materials [94]. For the case of finite temperature, the phenomenon of temperature-induced and magnetic field-induced entanglement were predicted. The behaviour of the Wootters concurrence was extensively discussed, developing the interest in thermal entanglement and magnetic entanglement, as called in pioneering work, Ref. [25] exposing the interplay of the temperature and magnetic field when influencing the entanglement of naturally occurring thermal quantum states in magnetic nanoclusters.

The system in question was inspired by synthesis of Cu-based cluster molecular magnets containing spins 1/2 [77] and Co-based structures [74] built of higher spins. Our selection of cluster containing localized spins S=1/2 (equivalent to qubits) was additionally justified by expectation of most pronounced quantum behaviour for the lowest possible spin. However, the extension of the study to higher spins would further augment the parameter space, leading to enriched phase diagram and possibility to explore entanglement for more general qudit case. Another direction of extending the study would involve incorporation of intercluster couplings to correlate the model better to cluster molecular magnets in which such couplings occur (see a general model study [95] or particular examples of coupled dimers [96,97]).

## Data Availability

The data presented in this study are available on reasonable request from the corresponding author.

## Sample Availability

No samples were used in this study.

## Appendix A. Complete Set of the Eigenvalues and Eigenstates of the Cluster Hamiltonian

The appendix contains the complete set of eigenstates of cluster Hamiltonian given by Equation (1) with relevant eigenenergies and sets of quantum numbers $S_{T}$, $S_{T,z}$ and *s* [related to the eigenenergies by Equation (2)]. The states are listed in two forms: Table A1 contains the eigenstates written in the basis using only spin-up and spin-down states for single spins, whereas Table A2 manifestly emphasizes division of the system to central spin and two dimers using Bell states for spin pairs if possible.

**Table A1:** Eigenenergies and eigenstates of the Hamiltonian [Equation (1)] with quantum numbers.

$S_{T},\;S_{T,z},\;s$	Eigenenergy	Eigenstate
$\frac{5}{2},+\frac{5}{2},2$	$-J_{1}-\frac{3}{2}J_{2}-\frac{5}{2}H$	$|\psi_{\frac{5}{2},+\frac{5}{2},2}\rangle =|↑↑↑↑↑\rangle$
$\frac{5}{2},+\frac{3}{2},2$	$-J_{1}-\frac{3}{2}J_{2}-\frac{3}{2}H$	$|\psi_{\frac{5}{2},+\frac{3}{2},2}\rangle =\frac{1}{\sqrt{5}}\left(|↑↑↑↑↓\rangle +|↑↑↑↓↑\rangle +|↑↑↓↑↑\rangle +|↑↓↑↑↑\rangle +|↓↑↑↑↑\rangle \right)$
$\frac{5}{2},-\frac{1}{2},2$	$-J_{1}-\frac{3}{2}J_{2}-\frac{1}{2}H$	$|\psi_{\frac{5}{2},+\frac{1}{2},2}\rangle =\frac{1}{\sqrt{10}}\left(|↑↑↑↓↓\rangle +|↑↑↓↑↓\rangle +|↑↓↑↑↓\rangle +|↓↑↑↑↓\rangle +|↑↑↓↓↑\rangle +|↑↓↑↓↑\rangle \right.$
		$\;\;\;\;\;\;\;\;\;\;\;\;\;\;\;\;\;\;\;\;\;\;\;\;\;\;\;\;\;\;\;\;\;\;\;\;\;\;\;\;\;\left.+|↓↑↑↓↑\rangle +|↑↓↓↑↑\rangle +|↓↑↓↑↑\rangle +|↓↓↑↑↑\rangle \right)$
$\frac{5}{2},-\frac{1}{2},2$	$-J_{1}-\frac{3}{2}J_{2}+\frac{1}{2}H$	$|\psi_{\frac{5}{2},-\frac{1}{2},2}\rangle =\frac{1}{\sqrt{10}}\left(|↓↓↓↑↑\rangle +|↓↓↑↓↑\rangle +|↓↑↓↓↑\rangle +|↑↓↓↓↑\rangle +|↓↓↑↑↓\rangle +|↓↑↓↑↓\rangle \right.$
		$\;\;\;\;\;\;\;\;\;\;\;\;\;\;\;\;\;\;\;\;\;\;\;\;\;\;\;\;\;\;\;\;\;\;\;\;\;\;\;\;\;\left.+|↑↓↓↑↓\rangle +|↓↑↑↓↓\rangle +|↑↓↑↓↓\rangle +|↑↑↓↓↓\rangle \right)$
$\frac{5}{2},-\frac{3}{2},2$	$-J_{1}-\frac{3}{2}J_{2}+\frac{3}{2}H$	$|\psi_{\frac{5}{2},-\frac{3}{2},2}\rangle =\frac{1}{\sqrt{5}}\left(|↓↓↓↓↑\rangle +|↓↓↓↑↓\rangle +|↓↓↑↓↓\rangle +|↓↑↓↓↓\rangle +|↑↓↓↓↓\rangle \right)$
$\frac{5}{2},-\frac{5}{2},2$	$-J_{1}-\frac{3}{2}J_{2}+\frac{5}{2}H$	$|\psi_{\frac{5}{2},-\frac{5}{2},2}\rangle =|↓↓↓↓↓\rangle$
$\frac{3}{2},+\frac{3}{2},2$	$\frac{3}{2}J_{1}-\frac{3}{2}J_{2}-\frac{3}{2}H$	$|\psi_{\frac{3}{2},+\frac{3}{2},2}\rangle =\frac{1}{2\sqrt{5}}\left(|↑↑↑↑↓\rangle +|↑↑↑↓↑\rangle +|↑↑↓↑↑\rangle +|↑↓↑↑↑\rangle -4|↓↑↑↑↑\rangle \right)$
$\frac{3}{2},+\frac{1}{2},2$	$\frac{3}{2}J_{1}-\frac{3}{2}J_{2}-\frac{1}{2}H$	$|\psi_{\frac{3}{2},+\frac{1}{2},2}\rangle =\frac{1}{2\sqrt{15}}\left(2|↑↑↑↓↓\rangle +2|↑↑↓↑↓\rangle +2|↑↑↓↓↑\rangle +2|↑↓↑↑↓\rangle +2|↑↓↑↓↑\rangle +2|↑↓↓↑↑\rangle \right.$
		$\;\;\;\;\;\;\;\;\;\;\;\;\;\;\;\;\;\;\;\;\;\;\;\;\;\;\;\;\;\;\;\;\;\;\;\;\;\;\;\;\;\;\;\left.-3|↓↑↑↑↓\rangle -3|↓↑↑↓↑\rangle -3|↓↑↓↑↑\rangle -3|↓↓↑↑↑\rangle \right)$
$\frac{3}{2},-\frac{1}{2},2$	$\frac{3}{2}J_{1}-\frac{3}{2}J_{2}+\frac{1}{2}H$	$|\psi_{\frac{3}{2},-\frac{1}{2},2}\rangle =\frac{1}{2\sqrt{15}}\left(2|↓↓↓↑↑\rangle +2|↓↓↑↓↑\rangle +2|↓↓↑↑↓\rangle +2|↓↑↓↓↑\rangle +2|↓↑↓↑↓\rangle +2|↓↑↑↓↓\rangle \right.$
		$\;\;\;\;\;\;\;\;\;\;\;\;\;\;\;\;\;\;\;\;\;\;\;\;\;\;\;\;\;\;\;\;\;\;\;\;\;\;\;\;\;\;\;\left.-3|↑↓↓↓↑\rangle -3|↑↓↓↑↓\rangle -3|↑↓↑↓↓\rangle -3|↑↑↓↓↓\rangle \right)$
$\frac{3}{2},-\frac{3}{2},2$	$\frac{3}{2}J_{1}-\frac{3}{2}J_{2}+\frac{3}{2}H$	$|\psi_{\frac{3}{2},-\frac{3}{2},2}\rangle =\frac{1}{2\sqrt{5}}\left(|↓↓↓↓↑\rangle +|↓↓↓↑↓\rangle +|↓↓↑↓↓\rangle +|↓↑↓↓↓\rangle -4|↑↓↓↓↓\rangle \right)$
$\frac{3}{2},+\frac{3}{2},1$	$-\frac{1}{2}J_{1}+\frac{1}{2}J_{2}-\frac{3}{2}H$	$|\psi_{\frac{3}{2},+\frac{3}{2},1}^{\left(1\right)}\rangle =\frac{1}{2}\left(|↑↑↑↑↓\rangle -|↑↑↑↓↑\rangle +|↑↑↓↑↑\rangle -|↑↓↑↑↑\rangle \right)$
		$|\psi_{\frac{3}{2},+\frac{3}{2},1}^{\left(2\right)}\rangle =\frac{1}{2}\left(|↑↑↑↑↓\rangle -|↑↑↑↓↑\rangle -|↑↑↓↑↑\rangle +|↑↓↑↑↑\rangle \right)$
		$|\psi_{\frac{3}{2},+\frac{3}{2},1}^{\left(3\right)}\rangle =\frac{1}{2}\left(|↑↑↑↑↓\rangle +|↑↑↑↓↑\rangle -|↑↑↓↑↑\rangle -|↑↓↑↑↑\rangle \right)$
$\frac{3}{2},+\frac{1}{2},1$	$-\frac{1}{2}J_{1}+\frac{1}{2}J_{2}-\frac{1}{2}H$	$|\psi_{\frac{3}{2},+\frac{1}{2},1}^{\left(1\right)}\rangle =\frac{1}{2\sqrt{3}}\left(|↓↓↑↑↑\rangle +|↓↑↓↑↑\rangle -|↓↑↑↓↑\rangle -|↓↑↑↑↓\rangle +2|↑↓↓↑↑\rangle -2|↑↑↑↓↓\rangle \right)$
		$|\psi_{\frac{3}{2},+\frac{1}{2},1}^{\left(2\right)}\rangle =\frac{1}{2\sqrt{3}}\left(|↓↓↑↑↑\rangle -|↓↑↓↑↑\rangle -|↓↑↑↓↑\rangle +|↓↑↑↑↓\rangle +2|↑↓↑↑↓\rangle -2|↑↑↓↓↑\rangle \right)$
		$|\psi_{\frac{3}{2},+\frac{1}{2},1}^{\left(3\right)}\rangle =\frac{1}{2\sqrt{3}}\left(|↓↓↑↑↑\rangle -|↓↑↓↑↑\rangle +|↓↑↑↓↑\rangle -|↓↑↑↑↓\rangle -2|↑↑↓↑↓\rangle +2|↑↓↑↓↑\rangle \right)$
$\frac{3}{2},-\frac{1}{2},1$	$-\frac{1}{2}J_{1}+\frac{1}{2}J_{2}+\frac{1}{2}H$	$|\psi_{\frac{3}{2},-\frac{1}{2},1}^{\left(3\right)}\rangle =\frac{1}{2\sqrt{3}}\left(|↑↑↓↓↓\rangle -|↑↓↑↓↓\rangle +|↑↓↓↑↓\rangle -|↑↓↓↓↑\rangle -2|↓↓↑↓↑\rangle +2|↓↑↓↑↓\rangle \right)$
		$|\psi_{\frac{3}{2},-\frac{1}{2},1}^{\left(2\right)}\rangle =\frac{1}{2\sqrt{3}}\left(|↑↑↓↓↓\rangle -|↑↓↑↓↓\rangle -|↑↓↓↑↓\rangle +|↑↓↓↓↑\rangle +2|↓↑↓↓↑\rangle -2|↓↓↑↑↓\rangle \right)$
		$|\psi_{\frac{3}{2},-\frac{1}{2},1}^{\left(1\right)}\rangle =\frac{1}{2\sqrt{3}}\left(|↑↑↓↓↓\rangle +|↑↓↑↓↓\rangle -|↑↓↓↑↓\rangle -|↑↓↓↓↑\rangle +2|↓↑↑↓↓\rangle -2|↓↓↓↑↑\rangle \right)$
$\frac{3}{2},-\frac{3}{2},1$	$-\frac{1}{2}J_{1}+\frac{1}{2}J_{2}+\frac{3}{2}H$	$|\psi_{\frac{3}{2},-\frac{3}{2},1}^{\left(3\right)}\rangle =\frac{1}{2}\left(|↓↓↓↓↑\rangle +|↓↓↓↑↓\rangle -|↓↓↑↓↓\rangle -|↓↑↓↓↓\rangle \right)$
		$|\psi_{\frac{3}{2},-\frac{3}{2},1}^{\left(2\right)}\rangle =\frac{1}{2}\left(|↓↓↓↓↑\rangle -|↓↓↓↑↓\rangle -|↓↓↑↓↓\rangle +|↓↑↓↓↓\rangle \right)$
		$|\psi_{\frac{3}{2},-\frac{3}{2},1}^{\left(1\right)}\rangle =\frac{1}{2}\left(|↓↓↓↓↑\rangle -|↓↓↓↑↓\rangle +|↓↓↑↓↓\rangle -|↓↑↓↓↓\rangle \right)$
$\frac{1}{2},+\frac{1}{2},1$	$J_{1}+\frac{1}{2}J_{2}-\frac{1}{2}H$	$|\psi_{\frac{1}{2},+\frac{1}{2},1}^{\left(1\right)}\rangle =\frac{1}{\sqrt{6}}\left(|↓↓↑↑↑\rangle -|↓↑↓↑↑\rangle +|↓↑↑↓↑\rangle -|↓↑↑↑↓\rangle -|↑↓↑↓↑\rangle +|↑↑↓↑↓\rangle \right)$
		$|\psi_{\frac{1}{2},+\frac{1}{2},1}^{\left(2\right)}\rangle =\frac{1}{\sqrt{6}}\left(|↓↓↑↑↑\rangle +|↓↑↓↑↑\rangle -|↓↑↑↓↑\rangle +|↓↑↑↑↓\rangle -|↑↓↓↑↑\rangle +|↑↑↑↓↓\rangle \right)$
		$|\psi_{\frac{1}{2},+\frac{1}{2},1}^{\left(3\right)}\rangle =\frac{1}{\sqrt{6}}\left(|↓↓↑↑↑\rangle -|↓↑↓↑↑\rangle -|↓↑↑↓↑\rangle +|↓↑↑↑↓\rangle -|↑↓↑↑↓\rangle +|↑↑↓↓↑\rangle \right)$
$\frac{1}{2},-\frac{1}{2},1$	$J_{1}+\frac{1}{2}J_{2}+\frac{1}{2}H$	$|\psi_{\frac{1}{2},-\frac{1}{2},1}^{\left(3\right)}\rangle =\frac{1}{\sqrt{6}}\left(|↑↑↓↓↓\rangle -|↑↓↑↓↓\rangle -|↑↓↓↑↓\rangle +|↑↓↓↓↑\rangle -|↓↑↓↓↑\rangle +|↓↓↑↑↓\rangle \right)$
		$|\psi_{\frac{1}{2},-\frac{1}{2},1}^{\left(2\right)}\rangle =\frac{1}{\sqrt{6}}\left(|↑↑↓↓↓\rangle +|↑↓↑↓↓\rangle -|↑↓↓↑↓\rangle +|↑↓↓↓↑\rangle -|↓↑↑↓↓\rangle +|↓↓↓↑↑\rangle \right)$
		$|\psi_{\frac{1}{2},-\frac{1}{2},1}^{\left(1\right)}\rangle =\frac{1}{\sqrt{6}}\left(|↑↑↓↓↓\rangle -|↑↓↑↓↓\rangle +|↑↓↓↑↓\rangle -|↑↓↓↓↑\rangle -|↓↑↓↑↓\rangle +|↓↓↑↓↑\rangle \right)$
$\frac{1}{2},+\frac{1}{2},0$	$\frac{3}{2}J_{2}-\frac{1}{2}H$	$|\psi_{\frac{1}{2},+\frac{1}{2},0}^{\left(1\right)}\rangle =\frac{1}{2}\left(|↑↑↓↑↓\rangle -|↑↑↓↓↑\rangle +|↑↓↑↓↑\rangle -|↑↓↑↑↓\rangle \right)$
		$|\psi_{\frac{1}{2},+\frac{1}{2},0}^{\left(2\right)}\rangle =\frac{1}{2\sqrt{3}}\left(2|↑↑↑↓↓\rangle -|↑↑↓↑↓\rangle -|↑↑↓↓↑\rangle -|↑↓↑↑↓\rangle -|↑↓↑↓↑\rangle +2|↑↓↓↑↑\rangle \right)$
$\frac{1}{2},-\frac{1}{2},0$	$\frac{3}{2}J_{2}+\frac{1}{2}H$	$|\psi_{\frac{1}{2},-\frac{1}{2},0}^{\left(2\right)}\rangle =\frac{1}{2\sqrt{3}}\left(2|↓↓↓↑↑\rangle -|↓↓↑↓↑\rangle -|↓↓↑↑↓\rangle -|↓↑↓↓↑\rangle -|↓↑↓↑↓\rangle +2|↓↑↑↓↓\rangle \right)$
		$|\psi_{\frac{1}{2},-\frac{1}{2},0}^{\left(1\right)}\rangle =\frac{1}{2}\left(|↓↓↑↓↑\rangle -|↓↓↑↑↓\rangle +|↓↑↓↑↓\rangle -|↓↑↓↓↑\rangle \right)$

**Table A2:** Eigenenergies and eigenstates of the Hamiltonian [Equation (1)] with quantum numbers, expressed in a basis emphasizing the system division into central spin and two dimers.

$S_{T},\;S_{T,z},\;s$	Eigenenergy	Eigenstate
$\frac{5}{2},+\frac{5}{2},2$	$-J_{1}-\frac{3}{2}J_{2}-\frac{5}{2}H$	$|\psi_{\frac{5}{2},+\frac{5}{2},2}\rangle =|↑\rangle |↑↑\rangle |↑↑\rangle$
$\frac{5}{2},+\frac{3}{2},2$	$-J_{1}-\frac{3}{2}J_{2}-\frac{3}{2}H$	$|\psi_{\frac{5}{2},+\frac{3}{2},2}\rangle =\sqrt{\frac{2}{5}}|↑\rangle \left(|↑↑\rangle |\psi^{+}\rangle +|\psi^{+}\rangle |↑↑\rangle \right)+\frac{1}{\sqrt{5}}|↓\rangle |↑↑\rangle |↑↑\rangle$
$\frac{5}{2},+\frac{1}{2},2$	$-J_{1}-\frac{3}{2}J_{2}-\frac{1}{2}H$	$|\psi_{\frac{5}{2},+\frac{1}{2},2}\rangle =\frac{1}{\sqrt{10}}|↑\rangle \left(|\phi^{+}\rangle |\phi^{+}\rangle -|\phi^{-}\rangle |\phi^{-}\rangle +2|\psi^{+}\rangle |\psi^{+}\rangle \right)+\frac{1}{\sqrt{5}}|↓\rangle \left(|↑↑\rangle |\psi^{+}\rangle +|\psi^{+}\rangle |↑↑\rangle \right)$
$\frac{5}{2},-\frac{1}{2},2$	$-J_{1}-\frac{3}{2}J_{2}+\frac{1}{2}H$	$|\psi_{\frac{5}{2},-\frac{1}{2},2}\rangle =\frac{1}{\sqrt{10}}|↓\rangle \left(|\phi^{+}\rangle |\phi^{+}\rangle -|\phi^{-}\rangle |\phi^{-}\rangle +2|\psi^{+}\rangle |\psi^{+}\rangle \right)+\frac{1}{\sqrt{5}}|↑\rangle \left(|↓↓\rangle |\psi^{+}\rangle +|\psi^{+}\rangle |↓↓\rangle \right)$
$\frac{5}{2},-\frac{3}{2},2$	$-J_{1}-\frac{3}{2}J_{2}+\frac{3}{2}H$	$|\psi_{\frac{5}{2},-\frac{3}{2},2}\rangle =\sqrt{\frac{2}{5}}|↓\rangle \left(|↓↓\rangle |\psi^{+}\rangle +|\psi^{+}\rangle |↓↓\rangle \right)+\frac{1}{\sqrt{5}}|↑\rangle |↓↓\rangle |↓↓\rangle$
$\frac{5}{2},-\frac{5}{2},2$	$-J_{1}-\frac{3}{2}J_{2}+\frac{5}{2}H$	$|\psi_{\frac{5}{2},-\frac{5}{2},2}\rangle =|↓\rangle |↓↓\rangle |↓↓\rangle$
$\frac{3}{2},+\frac{3}{2},2$	$\frac{3}{2}J_{1}-\frac{3}{2}J_{2}-\frac{3}{2}H$	$|\psi_{\frac{3}{2},+\frac{3}{2},2}\rangle =\frac{1}{\sqrt{10}}|↑\rangle \left(|↑↑\rangle |\psi^{+}\rangle +|\psi^{+}\rangle |↑↑\rangle \right)-\frac{2}{\sqrt{5}}|↓\rangle |↑↑\rangle |↑↑\rangle$
$\frac{3}{2},+\frac{1}{2},2$	$\frac{3}{2}J_{1}-\frac{3}{2}J_{2}-\frac{1}{2}H$	$|\psi_{\frac{3}{2},+\frac{1}{2},2}\rangle =\frac{1}{\sqrt{15}}|↑\rangle \left(|\phi^{+}\rangle |\phi^{+}\rangle -|\phi^{-}\rangle |\phi^{-}\rangle +2|\psi^{+}\rangle |\psi^{+}\rangle \right)-\frac{3}{\sqrt{30}}|↓\rangle \left(|↑↑\rangle |\psi^{+}\rangle +|\psi^{+}\rangle |↑↑\rangle \right)$
$\frac{3}{2},-\frac{1}{2},2$	$\frac{3}{2}J_{1}-\frac{3}{2}J_{2}+\frac{1}{2}H$	$|\psi_{\frac{3}{2},-\frac{1}{2},2}\rangle =\frac{1}{\sqrt{15}}|↓\rangle \left(|\phi^{+}\rangle |\phi^{+}\rangle -|\phi^{-}\rangle |\phi^{-}\rangle +2|\psi^{+}\rangle |\psi^{+}\rangle \right)-\frac{3}{\sqrt{30}}|↑\rangle \left(|↓↓\rangle |\psi^{+}\rangle +|\psi^{+}\rangle |↓↓\rangle \right)$
$\frac{3}{2},-\frac{3}{2},2$	$\frac{3}{2}J_{1}-\frac{3}{2}J_{2}+\frac{3}{2}H$	$|\psi_{\frac{3}{2},-\frac{3}{2},2}\rangle =\frac{1}{\sqrt{10}}|↓\rangle \left(|↓↓\rangle |\psi^{+}\rangle +|\psi^{+}\rangle |↓↓\rangle \right)-\frac{2}{\sqrt{5}}|↑\rangle |↓↓\rangle |↓↓\rangle$
$\frac{3}{2},+\frac{3}{2},1$	$-\frac{1}{2}J_{1}+\frac{1}{2}J_{2}-\frac{3}{2}H$	$|\psi_{\frac{3}{2},+\frac{3}{2},1}^{\left(1\right)}\rangle =\frac{1}{\sqrt{2}}|↑\rangle \left(|↑↑\rangle |\psi^{-}\rangle +|\psi^{-}\rangle |↑↑\rangle \right)$
		$|\psi_{\frac{3}{2},+\frac{3}{2},1}^{\left(2\right)}\rangle =\frac{1}{\sqrt{2}}|↑\rangle \left(|↑↑\rangle |\psi^{-}\rangle -|\psi^{-}\rangle |↑↑\rangle \right)$
		$|\psi_{\frac{3}{2},+\frac{3}{2},1}^{\left(3\right)}\rangle =\frac{1}{\sqrt{2}}|↑\rangle \left(|↑↑\rangle |\psi^{+}\rangle -|\psi^{+}\rangle |↑↑\rangle \right)$
$\frac{3}{2},+\frac{1}{2},1$	$-\frac{1}{2}J_{1}+\frac{1}{2}J_{2}-\frac{1}{2}H$	$|\psi_{\frac{3}{2},+\frac{1}{2},1}^{\left(1\right)}\rangle =\frac{1}{\sqrt{6}}|↓\rangle \left(|\psi^{+}\rangle |↑↑\rangle -|↑↑\rangle |\psi^{+}\rangle \right)-\frac{1}{\sqrt{3}}|↑\rangle \left(|\phi^{-}\rangle |\phi^{+}\rangle -|\phi^{+}\rangle |\phi^{-}\rangle \right)$
		$|\psi_{\frac{3}{2},+\frac{1}{2},1}^{\left(2\right)}\rangle =\frac{1}{\sqrt{6}}|↓\rangle \left(|\psi^{-}\rangle |↑↑\rangle -|↑↑\rangle |\psi^{-}\rangle \right)+\frac{1}{\sqrt{3}}|↑\rangle \left(|\psi^{-}\rangle |\psi^{+}\rangle -|\psi^{+}\rangle |\psi^{-}\rangle \right)$
		$|\psi_{\frac{3}{2},+\frac{1}{2},1}^{\left(3\right)}\rangle =\frac{1}{\sqrt{6}}|↓\rangle \left(|\psi^{-}\rangle |↑↑\rangle +|↑↑\rangle |\psi^{-}\rangle \right)+\frac{1}{\sqrt{3}}|↑\rangle \left(|\psi^{-}\rangle |\psi^{+}\rangle +|\psi^{+}\rangle |\psi^{-}\rangle \right)$
$\frac{3}{2},-\frac{1}{2},1$	$-\frac{1}{2}J_{1}+\frac{1}{2}J_{2}+\frac{1}{2}H$	$|\psi_{\frac{3}{2},-\frac{1}{2},1}^{\left(3\right)}\rangle =\frac{1}{\sqrt{6}}|↑\rangle \left(|\psi^{-}\rangle |↓↓\rangle +|↓↓\rangle |\psi^{-}\rangle \right)+\frac{1}{\sqrt{3}}|↓\rangle \left(|\psi^{-}\rangle |\psi^{+}\rangle +|\psi^{+}\rangle |\psi^{-}\rangle \right)$
		$|\psi_{\frac{3}{2},-\frac{1}{2},1}^{\left(2\right)}\rangle =\frac{1}{\sqrt{6}}|↑\rangle \left(|\psi^{-}\rangle |↓↓\rangle -|↓↓\rangle |\psi^{-}\rangle \right)+\frac{1}{\sqrt{3}}|↓\rangle \left(|\psi^{-}\rangle |\psi^{+}\rangle -|\psi^{+}\rangle |\psi^{-}\rangle \right)$
		$|\psi_{\frac{3}{2},-\frac{1}{2},1}^{\left(1\right)}\rangle =\frac{1}{\sqrt{6}}|↑\rangle \left(|\psi^{+}\rangle |↓↓\rangle -|↓↓\rangle |\psi^{+}\rangle \right)+\frac{1}{\sqrt{3}}|↓\rangle \left(|\phi^{-}\rangle |\phi^{+}\rangle -|\phi^{+}\rangle |\phi^{-}\rangle \right)$
$\frac{3}{2},-\frac{3}{2},1$	$-\frac{1}{2}J_{1}+\frac{1}{2}J_{2}+\frac{3}{2}H$	$|\psi_{\frac{3}{2},-\frac{3}{2},1}^{\left(3\right)}\rangle =\frac{1}{\sqrt{2}}|↓\rangle \left(|↓↓\rangle |\psi^{+}\rangle -|\psi^{+}\rangle |↓↓\rangle \right)$
		$|\psi_{\frac{3}{2},-\frac{3}{2},1}^{\left(2\right)}\rangle =\frac{1}{\sqrt{2}}|↓\rangle \left(|↓↓\rangle |\psi^{-}\rangle -|\psi^{-}\rangle |↓↓\rangle \right)$
		$|\psi_{\frac{3}{2},-\frac{3}{2},1}^{\left(1\right)}\rangle =\frac{1}{\sqrt{2}}|↓\rangle \left(|↓↓\rangle |\psi^{-}\rangle +|\psi^{-}\rangle |↓↓\rangle \right)$
$\frac{1}{2},+\frac{1}{2},1$	$J_{1}+\frac{1}{2}J_{2}-\frac{1}{2}H$	$|\psi_{\frac{1}{2},+\frac{1}{2},1}^{\left(1\right)}\rangle =\frac{1}{\sqrt{6}}|↑\rangle \left(|\psi^{+}\rangle |\psi^{-}\rangle +|\psi^{-}\rangle |\psi^{+}\rangle \right)-\frac{1}{\sqrt{3}}|↓\rangle \left(|↑↑\rangle |\psi^{-}\rangle +|\psi^{-}\rangle |↑↑\rangle \right)$
		$|\psi_{\frac{1}{2},+\frac{1}{2},1}^{\left(2\right)}\rangle =\frac{1}{\sqrt{6}}|↑\rangle \left(|\psi^{+}\rangle |\psi^{-}\rangle +|\psi^{-}\rangle |\psi^{+}\rangle \right)+\frac{1}{\sqrt{3}}|↓\rangle \left(|↑↑\rangle |\psi^{-}\rangle -|\psi^{-}\rangle |↑↑\rangle \right)$
		$|\psi_{\frac{1}{2},+\frac{1}{2},1}^{\left(3\right)}\rangle =\frac{1}{\sqrt{6}}|↑\rangle \left(|\psi^{+}\rangle |\psi^{-}\rangle +|\psi^{-}\rangle |\psi^{+}\rangle \right)-\frac{1}{\sqrt{3}}|↓\rangle \left(|↑↑\rangle |\psi^{+}\rangle -|\psi^{+}\rangle |↑↑\rangle \right)$
$\frac{1}{2},-\frac{1}{2},1$	$J_{1}+\frac{1}{2}J_{2}+\frac{1}{2}H$	$|\psi_{\frac{1}{2},-\frac{1}{2},1}^{\left(3\right)}\rangle =\frac{1}{\sqrt{6}}|↓\rangle \left(|\psi^{+}\rangle |\psi^{-}\rangle +|\psi^{-}\rangle |\psi^{+}\rangle \right)+\frac{1}{\sqrt{3}}|↑\rangle \left(|↓↓\rangle |\psi^{+}\rangle -|\psi^{+}\rangle |↓↓\rangle \right)$
		$|\psi_{\frac{1}{2},-\frac{1}{2},1}^{\left(2\right)}\rangle =\frac{1}{\sqrt{6}}|↓\rangle \left(|\psi^{+}\rangle |\psi^{-}\rangle +|\psi^{-}\rangle |\psi^{+}\rangle \right)+\frac{1}{\sqrt{3}}|↑\rangle \left(|↓↓\rangle |\psi^{-}\rangle -|\psi^{-}\rangle |↓↓\rangle \right)$
		$|\psi_{\frac{1}{2},-\frac{1}{2},1}^{\left(1\right)}\rangle =\frac{1}{\sqrt{6}}|↓\rangle \left(|\psi^{+}\rangle |\psi^{-}\rangle +|\psi^{-}\rangle |\psi^{+}\rangle \right)-\frac{1}{\sqrt{3}}|↑\rangle \left(|↑↑\rangle |\psi^{-}\rangle +|\psi^{-}\rangle |↑↑\rangle \right)$
$\frac{1}{2},+\frac{1}{2},0$	$\frac{3}{2}J_{2}-\frac{1}{2}H$	$|\psi_{\frac{1}{2},+\frac{1}{2},0}^{\left(1\right)}\rangle =|↑\rangle |\psi^{-}\rangle |\psi^{-}\rangle$
		$|\psi_{\frac{1}{2},+\frac{1}{2},0}^{\left(2\right)}\rangle =\frac{1}{\sqrt{3}}|↑\rangle \left(|\phi^{+}\rangle |\phi^{+}\rangle -|\phi^{-}\rangle |\phi^{-}\rangle -|\psi^{+}\rangle |\psi^{+}\rangle \right)$
$\frac{1}{2},-\frac{1}{2},0$	$\frac{3}{2}J_{2}+\frac{1}{2}H$	$|\psi_{\frac{1}{2},-\frac{1}{2},0}^{\left(2\right)}\rangle =\frac{1}{\sqrt{3}}|↓\rangle \left(|\phi^{+}\rangle |\phi^{+}\rangle -|\phi^{-}\rangle |\phi^{-}\rangle -|\psi^{+}\rangle |\psi^{+}\rangle \right)$
		$|\psi_{\frac{1}{2},-\frac{1}{2},0}^{\left(1\right)}\rangle =|↓\rangle |\psi^{-}\rangle |\psi^{-}\rangle$
